# Hybrid ^18^F-florbetapir PET/MRI for assessing myelin recovery in GFAP-A patients

**DOI:** 10.1515/tnsci-2022-0223

**Published:** 2022-06-09

**Authors:** Huanyu Meng, Shuyu Zheng, Shaicun Yuan, Qinming Zhou, Yining Gao, You Ni, Lu He, Dou Yin, Min Zhang, Sheng Chen

**Affiliations:** Department of Neurology, Ruijin Hospital, Shanghai Jiaotong University School of Medicine, Shanghai, 200025, China; Department of Neurosurgery, The Second Affiliated Hospital, Zhejiang University, Hangzhou, Zhejiang Province, 310003, China; Department of Neurology, Binhai County People’s Hospital, Yancheng, Jiangsu Province, 224500, China; Department of Nuclear Medicine, Ruijin Hospital, Shanghai Jiao Tong University School of Medicine, Shanghai, 200025, China; Co-innovation Center of Neuroregeneration, Nantong University, Nantong 226001, China

**Keywords:** glial fibrillary acidic protein, neuroinflammation, ^18^F-florbetapir PET/MRI, autoimmune encephalitis, myelin recovery

## Abstract

Glial fibrillary acidic protein astrocytopathy (GFAP-A) is a rare autoimmune disease of the central nervous system that was newly reported in 2016. Previous studies have speculated that the pathological mechanism and clinical outcome of GFAP-A lie in the demyelination of the central nervous system, but due to the limitations of MR, this conclusion has not been further confirmed from the perspective of neuroimaging. A non-invasive, quantitative measurement of demyelination would be clinically valuable, given its critical role in mediating GFAP-A. Here, we report a case in which we use ^18^F-florbetapir positron emission tomography-magnetic resonance imaging (PET/MRI) to evaluate myelin recovery with follow-up in the patient with GFAP-A. Our patient displayed a decreased uptake of PET tracer ^18^F-florbetapir in the brain lesions and lower distribution volume ratio in the damaged white matter lesions compared to the normal-appearing white matter, indicating significant intracranial demyelination. After treatment, the ^18^F-florbetapir PET/MRI examination showed a significant increase in the uptake of ^18^F-florbetapir in the brain lesions, along with a reduced Expanded Disability Status Scale score. Although only a small number of patients have been validated, this case first reported ^18^F-florbetapir PET/MRI could quantitatively and non-invasively assess the myelin recovery in GFAP-A patients, which may lead to improvements in the early diagnosis and long-term prognosis.

## Abbreviations

GFAP-A: glial fibrillary acidic protein astrocytopathy
IgG: immunoglobulin G
CSF: cerebrospinal fluid
CNS: central nervous system
DVR: distribution volume ratio
DWM: damaged white matter
NAWM: normal-appearing white matter
PET: positron emission tomography
MRI: magnetic resonance imaging
NGS: next generation sequencing
EDSS: Expanded Disability Status Scale
MS: multiple sclerosis
NMOSD: neuromyelitis optica spectrum disorders
AQP4: aquaporin-4
anti-MBP antibody: anti-myelin basic protein antibody
ADEM: acute disseminated encephalomyelitis
TSPO: the 18 kDa translocator protein

## 1 Introduction

Autoimmune glial fibrillary acidic protein (GFAP) astrocytopathy (GFAP-A) is a rare inflammatory central nervous system (CNS) disorder first reported in 2016 [1]. The clinical manifestations include fever, headache, encephalopathy, myelitis and abnormal vision [2]. Although GFAP autoantibody has been reported in the cerebrospinal fluid (CSF) and serum of patients with GFAP-A, it is still controversial whether GFAP antibody plays a key role in the demyelination of GFAP-A [1,2]. It is well known that GFAP is essential to the long-term maintenance of CNS myelination and blood–brain barrier integrity, while its destruction mediated by GFAP antibody may lead to CNS demyelination and astroglial cell activation [3]. Therefore, the degree of demyelination in GFAP-A is compatible with neurological dysfunction and long-term prognosis [4]. But this conclusion is not widely confirmed. The evaluation of myelin recovery in the CNS has great significance in the early diagnosis and prognosis evaluation of GFAP-A patients. But until now, apart from the autopsy after the patient died, there is a rare non-invasive examination that can specifically and quantitatively reflect the degree of the patient’s myelin loss or recovery in the brain and spinal cord [5].

Magnetic resonance imaging (MRI) is a preferred radiological modality in the early diagnosis of GFAP-A, with a typical pattern of gadolinium enhancement in brain MRI, and longitudinally extensive T2 hyperintensities with central cord enhancement in spine MRI [6]. Although conventional MRI can evaluate the location and size of GFAP-A, it cannot provide a quantitative assessment of myelin recovery. Recently, several advanced MRI techniques are introduced to the assessment in the demyelination of CNS, including magnetization transfer and diffusion-weighted imaging, while these techniques are less specific than tracer-based positron emission tomography (PET) toward myelin assessment [7,8,9,10]. ^18^F-Florbetapir, originally applied to the diagnosis of Alzheimer’s disease, was subsequently proved for their potential use in myelin imaging [11,12,13]. Our recent studies showed that amyloid PET tracer ^18^F-florbetapir bound to demyelinated lesions could monitor acute disseminated encephalomyelitis [13]. Nevertheless, the ability of ^18^F-florbetapir PET for assessing GFAP-A has not yet been investigated. Compared to conventional MRI which could reliably locate the lesion, a hybrid PET/MRI could simultaneously obtain quantitative and locational information specifically for myelin states. Therefore, we investigated the potential use of hybrid PET/MRI with ^18^F-florbetapir for quantitative assessment of demyelination in the case of patients with GFAP-A.

## 2 Methods

A patient with a definite diagnosis of GFAP-A was enrolled with follow-up. The enrolled patient underwent clinical assessments, including clinical disability through the expanded disability status scale (EDSS) before PET/MRI scan at baseline.

Based on the method we used before, ^18^F-florbetapir PET/MRI was performed with a Biograph mMR system (Siemens, Erlangen, Germany) with a National Electrical Manufacturers PET resolution of 4.2 mm [13]. After intravenous injection of approximately 296 MBq of ^18^F-florbetapir, dynamic PET acquisition in list mode over 60 min was started immediately, considering that it was cost-effective for most patients to reach a plateau [13]. Image standardization and segmentation were performed using statistical parametric mapping software (SPM12, Wellcome Centre for Human Neuroimaging, University College London, UK). The PET image was reconstructed by a point spread function algorithm with 344 × 344 pixels, 4 iterations, 21 subsets and a filter with a full width at half maximum of 2 mm. Both PET and MRI images were first normalized to MNI152 space by SPM12. Then, we used Lesion Segmentation Tool toolbox as a lesion prediction algorithm for damaged white matter (DWM) lesion segmentation. Normal-appearing white matter (NAWM) in GFAP-A patients was derived by subtracting the DWM segmentation from the total white matter segmentation. According to the supervised clustering method, the normal gray matter was selected as a reference region [11]. The Logan graphical reference method was then applied at the voxel level to produce a parametric map of ^18^F-florbetapir binding measured as the distribution volume ratio (DVR).


**Ethical approval:** The research related to human use has complied with all the relevant national regulations, institutional policies and in accordance with the tenets of the Helsinki Declaration. Human research was carried out with approval of the Ethics Committee of Ruijin Hospital, Shanghai Jiao Tong University School of Medicine and with informed patient consent.

## 3 Case presentation

A 26-year-old female developed headache and fever up to 41°C, accompanied by rigors, chills and vomiting. She was treated with antibiotics and appropriate rehydration at a local hospital, but her symptoms were not alleviated. Subsequently, she experienced several instances of disturbance of consciousness, agitation and rambling. She was emergently referred to the department of infectious disease in our hospital. On admission, CSF examination showed normal intracranial pressure, elevated protein (1988.06 mg/L) and reduced glucose (2.18 mmol/L), but next-generation sequencing of bacteria and fungi was both negative. Screening for associated antibodies revealed positive CSF GFAP antibodies (titer of 1:32) and serum GFAP antibodies (1:10). Brain MRI showed multiple abnormal signals involving right temporal lobe, left paraventricular and left frontal lobe.

**Figure 1:**
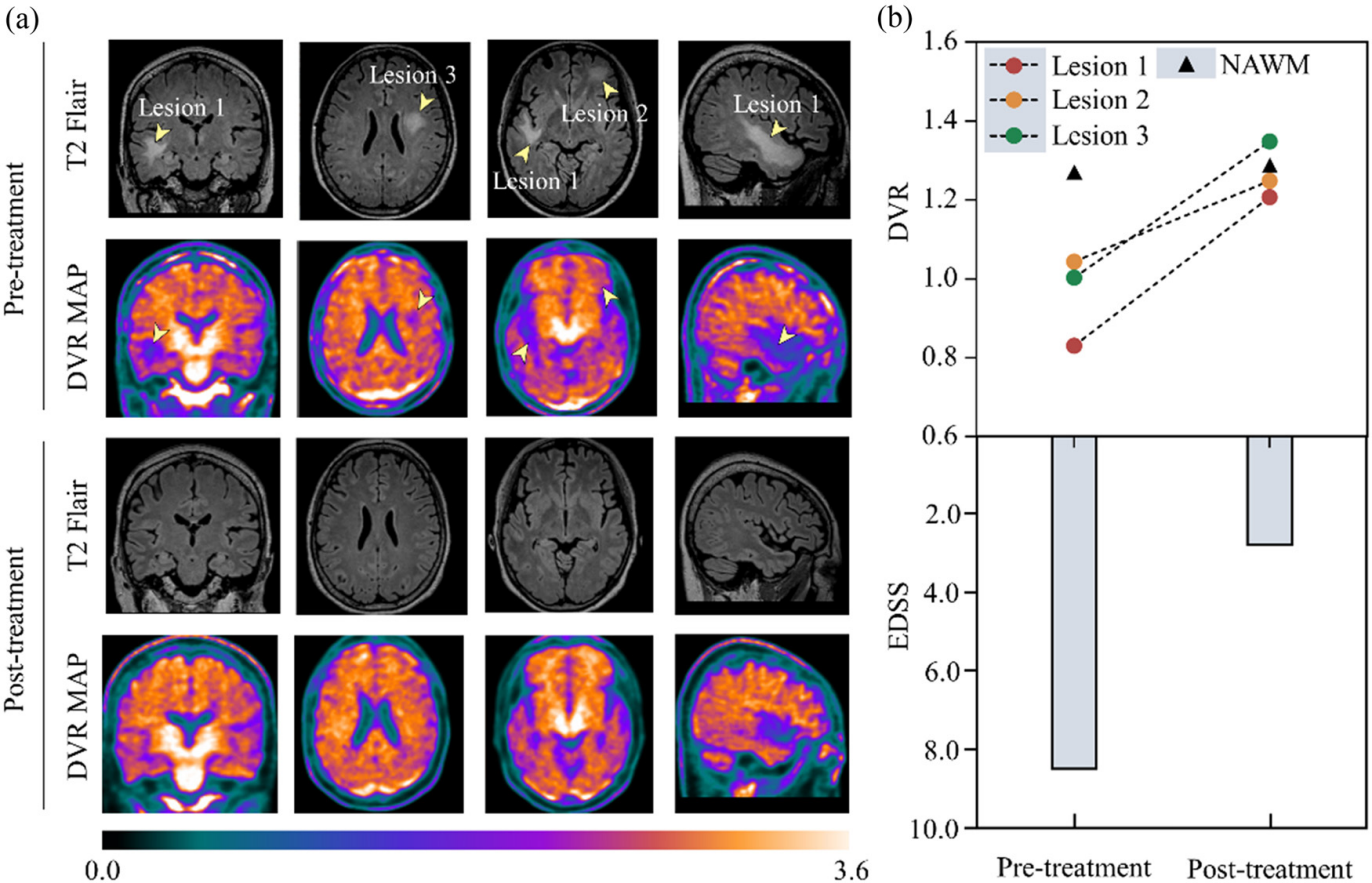
Demyelination and remyelination in three representative lesions on pre- and post-treatment ^18^F-florbetapir PET/MRI for the enrolled patient. (a) Pre-treatment PET/MRI showed multifocal hyperintense lesions (yellow arrowhead) with decreased ^18^F-florbetapir uptake (Lesion1 DVR = 0.83, Lesion2 DVR = 1.04, Lesion3 DVR = 1.00, and NAWM DVR = 1.21). After treatment, the MRI T2 Flair lesions at the same location were invisible, and the ^18^F-florbetapir uptake of the lesion was returned to a level equivalent to NAWM ^18^F-florbetapir uptake (Lesion1 DVR = 1.21, Lesion2 DVR = 1.25, Lesion3 DVR = 1.35, NAWM DVR = 1.24). (b) Along with the increase in ^18^F-florbetapir uptake in the three brain lesions, the patient’s clinical symptoms were also alleviated, with EDSS dropping from 8.5 to 2.5 with follow-up. DVR: distribution volume ratio; NAWM: normal-appearing white matter; EDSS: Expanded Disability Status Scale; MRI: magnetic resonance imaging.

In order to clarify the relationship between the location and scope of demyelinating lesions in the patient’s brain, we performed ^18^F-florbetapir PET/MRI. The decrease in the uptake of PET tracer ^18^F-florbetapir was found in the brain lesions on pre-treatment patient’s PET/MRI. Parallelly, T2 Flair MRI demonstrated multifocal hyperintense lesions (Figure 1a). Based on the method we introduced before, we chose the DVR for quantitatively measurement of the tracer uptake level [8,9]. We found the lower DVR in the three DWM lesions (Figure 1a, white arrow, Table 1, DVR = 0.96 ± 0.09), compared to the normal-appearing white matter (NAWM, DVR = 1.21), indicating significant intracranial demyelination.

**Table 1:** PET/MRI quantitative parameters of the enrolled patient

Lesions	Distance (mm)	Volume (mm^3^)	DVR	
			Pre-treatment	Post-treatment
Lesion 1	300	4,860	0.83	1.21
Lesion 2	480	1144.12	1.04	1.25
Lesion 3	555	3071.25	1.00	1.35
Mean			0.96	1.27
STD			0.09	0.06
NAWM			1.21	1.24

NAWM: normal-appearing white matter.

After completing the PET/MRI examination, the patient was given methylprednisolone and intravenous immunoglobulin therapy, followed by oral prednisone and mycophenolate mofetil. After 3 months of continuous oral immunosuppressive therapy, the patient’s clinical symptoms improved significantly, with a 6-point decrease in EDSS score (from 8.5 to 2.5, Figure 1b). Meanwhile, the PET/MRI examination showed that the uptake of ^18^F-florbetapir in the brain lesions of the pre-treatment patient was significantly increased and then returned to a level equivalent to NAWM ^18^F-florbetapir uptake (Figure 1, Table 1). As the DVRs of the DWM lesions were increased, the head MR lesions of the patient had disappeared, along with the reduced EDSS score, suggesting that the changes in head MR lesions are parallel to changes in both demyelinating lesions in the brain and clinical myelin recovery (Figure 1).

## 4 Discussion

Here, we report a hybrid PET/MRI imaging tool with ^18^F-florbetapir for quantitative assessment of myelin recovery in patients with GFAP-A. Among autoimmune demyelinating disorders, studies have shown a close relationship between demyelination and clinical manifestation including encephalopathy, meningeal symptoms and opticospinal abnormality [5]. However, the role of demyelination in the pathogenesis of GFAP-A has not been widely established, and the correlation between demyelination and EDSS scores remains to be demonstrated. Of note, the neuroinflammation and demyelination signals in conventional MRI are mixed in CNS demyelination diseases including GFAP-A. Therefore, for the evaluation of myelin recovery, traditional MR cannot meet the clinical requirements for quantitative evaluation of myelin recovery in GFAP-A patients. Plenty of studies have demonstrated ^18^F-florbetapir could serve as a quantitative myelin indicator, with its decrease in DWM lesions compared to NAWM among multiple sclerosis (MS), neuromyelitis optica spectrum disorders, and acute disseminated encephalomyelitis (ADEM) patients [9,11,12,13]. Parallel to these results, our study first showed a close relationship between PET tracer ^18^F-florbetapir uptake and myelin recovery in GFAP-A patients.

In our study, we found the similar trend was identified for the myelin change (as measured by ^18^F-florbetapir DVR) and the shift in the clinical disability (as measured using EDSS). A previous study conducted by our group has demonstrated that the EDSS change and global myelin recovery were significantly correlated in patients with MS [13]. Consistently with this result, our present study suggests that ^18^F-florbetapir DVR could provide additional valuable myelin state information in astrocytic damage-associated disease.

Based on the application of PET/MRI with ^18^F-florbetapir in ADEM and MS, our results for the first time indicate this technique could quantitatively and non-invasively assess the myelin recovery in GFAP-A patients and helps to understand the pathological process of the disease. This new approach should pave the way for real-time *in situ* myelin evaluations in clinical practice, and it offers the technical prerequisites for early diagnosis and long-term prognosis of demyelination-related disease.

## References

[1] Fang B, McKeon A, Hinson SR, Kryzer TJ, Pittock SJ, Aksamit AJ, et al. Autoimmune glial fibrillary acidic protein astrocytopathy: a novel meningoencephalomyelitis. JAMA Neurol. 2016;73(11):1297–307. 10.1001/jamaneurol.2016.2549 27618707

[2] Flanagan EP, Hinson SR, Lennon VA, Fang B, Aksamit AJ, Morris PP, et al. Glial fibrillary acidic protein immunoglobulin G as biomarker of autoimmune astrocytopathy: Analysis of 102 patients. Ann Neurol. 2017;81(2):298–309. 10.1002/ana.24881 28120349

[3] Skripuletz T, Hackstette D, Bauer K, Gudi V, Pul R, Voss E, et al. Astrocytes regulate myelin clearance through recruitment of microglia during cuprizone-induced demyelination. Brain. 2013;136(Pt 1):147–67. 10.1093/brain/aws262 23266461

[4] Hardy TA, Reddel SW, Barnett MH, Palace J, Lucchinetti CF, Weinshenker BG. Atypical inflammatory demyelinating syndromes of the CNS. Lancet Neurol. 2016;15(9):967–81. 10.1016/S1474-4422(16)30043-6 27478954

[5] Kunchok A, Zekeridou A, McKeon A. Autoimmune glial fibrillary acidic protein astrocytopathy. Curr Opin Neurol. 2019;32(3):452–8. 10.1097/WCO.0000000000000676 PMC6522205 30724768

[6] Gravier-Dumonceau A, Ameli R, Rogemond V, Ruiz A, Joubert B, Muñiz-Castrillo S, et al. Glial Fibrillary Acidic Protein Autoimmunity: A French Cohort Study. Neurology. 2022;98(6):e653–68. 10.1212/WNL.0000000000013087 PMC8829963 34799461

[7] Chen JT, Collins DL, Atkins HL, Freedman MS, Arnold DL. Canadian MS/BMT Study Group. Magnetization transfer ratio evolution with demyelination and remyelination in multiple sclerosis lesions. Ann Neurol. 2008;63(2):254–62. 10.1002/ana.21302 18257039

[8] Talbott JF, Nout-Lomas YS, Wendland MF, Mukherjee P, Huie JR, Hess CP, et al. Diffusion-weighted magnetic resonance imaging characterization of white matter injury produced by axon-sparing demyelination and severe contusion spinal cord injury in rats. J Neurotrauma. 2016;33(10):929–42. 10.1089/neu.2015.4102 PMC4876499 26483094

[9] Stankoff B, Freeman L, Aigrot MS, Chardain A, Dollé F, Williams A, et al. Imaging central nervous system myelin by positron emission tomography in multiple sclerosis using [methyl-(1)(1)C]-2-(4’-methylaminophenyl)- 6-hydroxybenzothiazole. Ann Neurol. 2011;69(4):673–80. 10.1002/ana.22320 21337603

[10] Bodini B, Veronese M, García-Lorenzo D, Battaglini M, Poirion E, Chardain A, et al. Dynamic imaging of individual remyelination profiles in multiple sclerosis. Ann Neurol. 2016;79(5):726–38. 10.1002/ana.24620 PMC5006855 26891452

[11] Carotenuto A, Giordano B, Dervenoulas G, Wilson H, Veronese M, Chappell Z, et al. [(18)F]Florbetapir PET/MR imaging to assess demyelination in multiple sclerosis. Eur J Nucl Med Mol Imaging. 2020;47(2):366–78. 10.1007/s00259-019-04533-y PMC6974490 31637481

[12] Zhang M, Liu J, Li B, Chen S. (18)F-florbetapir PET/MRI for quantitatively monitoring demyelination and remyelination in acute disseminated encephalomyelitis. EJNMMI Res. 2019;9(1):96. 10.1186/s13550-019-0568-8 PMC6851275 31720882

[13] Zhang M, Ni Y, Zhou Q, He L, Meng H, Gao Y, et al. (18)F-florbetapir PET/MRI for quantitatively monitoring myelin loss and recovery in patients with multiple sclerosis: A longitudinal study. E Clinical Med. 2021;37:100982. 10.1016/j.eclinm.2021.100982 PMC8234356 34195586

